# Hyperuricemia and coronary heart disease mortality: a meta-analysis of prospective cohort studies

**DOI:** 10.1186/s12872-016-0379-z

**Published:** 2016-10-28

**Authors:** Tian Zuo, Xuehui Liu, Lu Jiang, Shuai Mao, Xin Yin, Liheng Guo

**Affiliations:** 1 The Second Clinical College of Guangzhou University of Chinese Medicine, Guangdong Provincial Hospital of Chinese Medicine, Guangzhou, 510120 People’s Republic of China; 2 Department of Critical Care Medicine, Guangdong Provincial Hospital of Chinese Medicine, Guangzhou, 510120 People’s Republic of China; 3 Department of Cardiology, Yichang Hospital of Chinese Medicine, Clinical Medical College of Chinese Medicine, China Three Gorges University, Yichang, 443000 People’s Republic of China

**Keywords:** Hyperuricemia, Coronary heart disease, Mortality, Meta-analysis

## Abstract

**Background:**

Hyperuricemia may be associated with an increased risk of coronary heart disease (CHD) mortality; however, the results from prospective studies are conflicting. The objective of this study was to assess the association between hyperuricemia and risk of CHD mortality by performing a meta-analysis.

**Methods:**

Pubmed and Embase were searched for relevant prospective cohort studies published until July 2015. Studies were included only if they reported data on CHD mortality related to hyperuricemia in a general population. The pooled adjusted relative risk (RR) was calculated using a random-effects model.

**Results:**

A total of 14 studies involving 341 389 adults were identified. Hyperuricemia was associated with an increased risk of CHD mortality (RR: 1.14; 95 % CI: 1.06–1.23) and all-cause mortality (RR: 1.20; 95 % CI: 1.13–1.28). For each increase of 1 mg/dl of serum uric acid (SUA), the overall risks of CHD and all-cause mortality increased by 20 and 9 %, respectively. According to the gender subgroup analyses, hyperuricemia increased the risk of CHD mortality in women (RR: 1.47; 95 % CI: 1.21–1.73) compared to men (RR: 1.10; 95 % CI: 1.00–1.19). The risk of all-cause mortality was greater in women.

**Conclusions:**

Hyperuricemia may modestly increase the risk of CHD and all-cause mortality. Future research is needed to determine whether urate–lowering therapy has beneficial effects for reducing CHD mortality.

**Electronic supplementary material:**

The online version of this article (doi:10.1186/s12872-016-0379-z) contains supplementary material, which is available to authorized users.

## Background

Coronary heart disease (CHD) is a severe threat to human health and has a high mortality rate. Many traditional risk factors for CHD have been identified, such as hyperlipidemia, hypertension, diabetes, and smoking. Serum uric acid (SUA), the end product of purine metabolism via an enzymatic reaction involving xanthine oxidase, has also been correlated with CHD by several studies [1–3]. However, because of controversial epidemiologic findings and the lack of consistent evidence, whether SUA is an independent and causal risk factor for CHD remains unknown [4–10].

Several observational studies [4, 8, 11, 12] demonstrated that elevated SUA has a predictive value for CHD risk and that hyperuricemia may be an important causal factor for CHD mortality. However, other studies [5, 6, 13–15] contradict this. Many factors may contribute to the conflicting conclusions. Subjectively, differences of the studied populations, sample size, length of follow-up, and methods of statistical analyses could influence the outcome. Objectively, known risk factors, such as age, gender, fat, weight index and other potential confounding factors, under- or over -estimate the association between hyperuricemia and the risk of related disease.

However, regardless of whether hyperuricemia is a causal risk factor for CHD mortality, several pathophysiological mechanisms have been postulated for their relationship. SUA was considered to be a major antioxidant in humans with possible beneficial anti-atherosclerotic effects in the early years. However, for patients with hyperuricemia, elevated SUA may have a more negative role by stimulating oxidative stress and causing endothelial dysfunction and inflammatory reactions [16]. Moreover, the formation of oxygen free radicals and platelet adhesiveness are also induced by hyperuricemia [17]. These observations may explain some direct or indirect associations between hyperuricemia and CHD.

A previous meta-analysis [18] suggested that hyperuricemia is associated with the risk of CHD mortality and that the association was stronger in women than men. This study has practical implications regarding the predication and prevention of CHD mortality and has been cited frequently. However, we found that several of the extracted data (RR or 95%CI) calculated in the meta-analysis differed from the original studies. These errors may alter the overall pooled results. In addition, several relevant prospective studies have been published since the previous meta-analysis was conducted. To accurately and comprehensively estimate the influence of hyperuricemia on CHD mortality in general populations, we performed an updated meta-analysis.

## Methods

### Literature search

We performed a comprehensive literature search in Pubmed and Embase for relevant prospective cohort studies assessing the association between hyperuricemia and CHD mortality. The search covered from the date of inception until July 2015, and there was no language restriction. The searched terms included hyperuricemia, uric acid, coronary disease, coronary heart disease, coronary artery disease, cardiac heart disease, cardiovascular disease, death and mortality. In addition, the reference lists of the selected articles were manually screened for potential studies. Our meta-analysis was conducted according to the checklist of Meta-analysis of Observational Studies in Epidemiology (MOOSE) [19].

### Study selection

Studies that satisfied the following criteria were included: 1) a prospective cohort study of adult subjects; 2) described the association between hyperuricemia and CHD mortality; 3) an inception cohort involving adults without CHD; and 4) reported adjusted risk estimates for CHD mortality, such as relative risk (RR) or hazard ratio (HR) with a 95 % confidence interval (95 % CI).

### Data extraction and quality assessment

Two authors independently extracted data from all of the included studies using a standardized Excel file. The following data were extracted from each study: first author, publication year, geographical location, sample size, gender, age, duration of follow-up, definition of hyperuricemia, outcome definition, adjusted risk estimates regarding CHD and all-cause mortality, and confounding variables. The primary outcome was the risk estimate for the association between hyperuricemia and CHD mortality. The quality of selected studies was evaluated using the Newcastle-Ottawa Scale [20]. The quality score of the cohort studies was calculated based on three components as follows: selection of the study groups (0–4 points), comparability of study groups (0–2 points), and ascertainment of the interest outcome (0–3 points). The score ranges from 0 to 9 points, with a higher score indicating better methodological quality. Disagreements were resolved by consensus.

### Statistical analysis

To standardize the unit of SUA of the included studies, we converted it from μmol/L to mg/dl by dividing by 59.48. If a study reported the association between hyperuricemia and CHD mortality according to an age- or SUA level-specific category, each was included in the meta-analysis. A pooled estimate of the adjusted RR was calculated using the DerSimonian and Laird random-effects model. Heterogeneity across studies was evaluated using the I^2^ statistic, which is a quantitative measure of inconsistency across studies. A stratified analysis by gender was conducted to assess the gender-related heterogeneity in the adjusted RR of CHD and all-cause mortality. If evident heterogeneity was present, a sensitivity analysis was conducted by omitting each study in turn to identify a potential source. To explore the impact of the study characteristics, such as gender, study region (Asia vs. non-Asia), duration of follow-up (≤10 years vs. >10 years), and sample size (<10,000 vs. >10,000), on the pooled RR, we would conduct a multivariate meta-regression analysis. But only the number of studies providing a same effect size was more than ten can the analysis be done according to the requirements of statistics recommended by the Cochrane Collaboration. Publication bias was assessed using both Begg’s test and Egger’s test. A two-tailed *p*–value < 0.05 was considered statistically significant. All statistical analyses were performed using Stata 12.0.

## Results

### Characteristics of the eligible studies

**Fig. 1:**
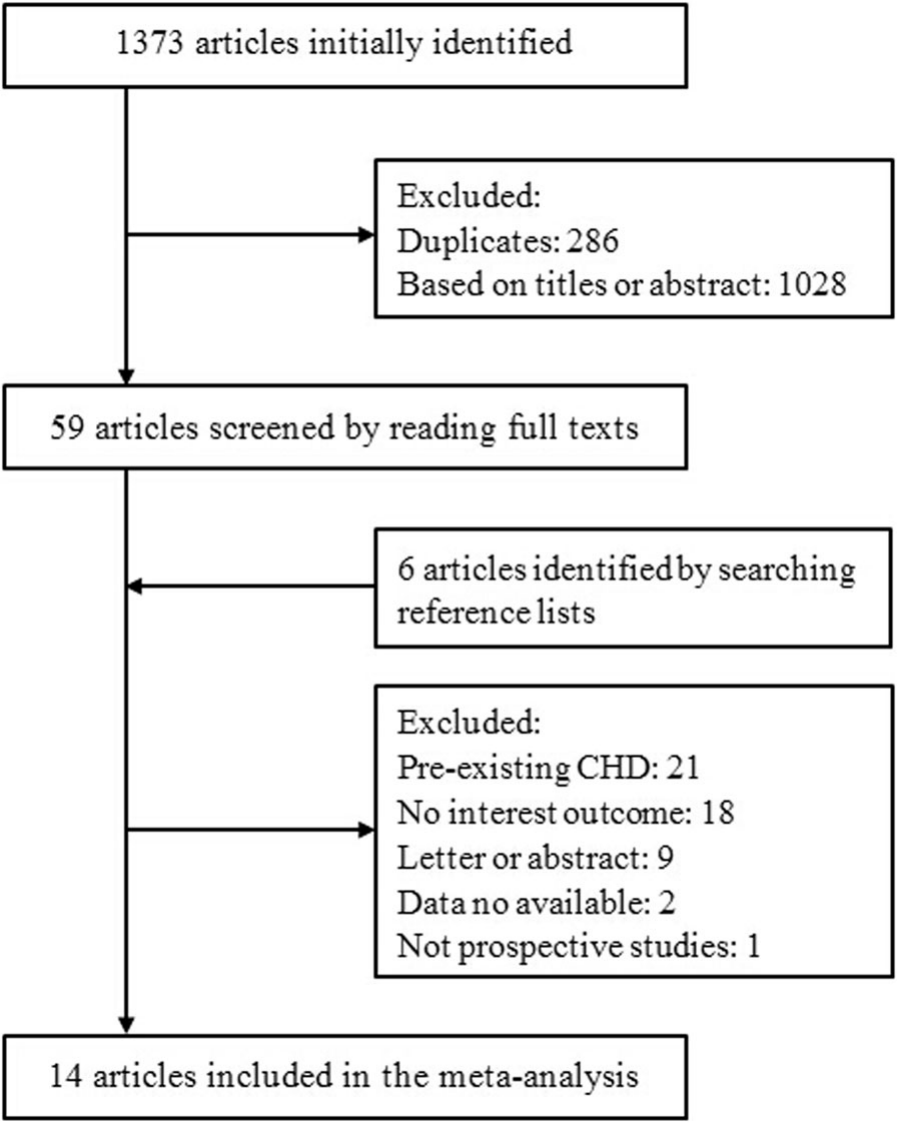
Search strategy

We retrieved 1373 articles with the initial literature search. Two-hundred-eighty-six articles were excluded because of duplicates. After screening the title or abstract, 1028 studies were excluded, and the remaining 59 were further identified by reading the full-text. According to the predefined inclusion criteria, 14 studies [21–34] enrolling 341 389 participants were included in the meta-analysis. Based on the reference lists of the included studies, we retrieved six potential studies, but none met our inclusion criteria. Figure 1 shows the detailed search strategy.

The characteristics of the included studies and their participants are summarized in Table 1. Of the 14 included studies, four were performed in the United States [21, 22, 29, 34], five in Europe [24, 26, 30, 31, 33] and five in Asia [22, 25, 27, 28, 32]. All except one [24] were written in English. The sample size of the studies ranged from 1198 [26] to 90 393 [32] participants. The duration of follow-up was between 5.4 [23] and 24.9 [27] years. Of these studies, seven [22, 24, 26, 27, 32–34] included both genders, five [23, 25, 28–30] included only men, and two [21, 31] included only women. The definition of hyperuricemia ranged from 5.6 to 7.0 mg/dl in men and from 5.4 to 7.0 mg/dl in women. Two studies reported the results of RR between hyperuricemia and CHD mortality based on the SUA level [23] and age [21] subgroup. Ten studies [23–25, 27–31, 33, 34] reported the association between the SUA level and CHD mortality based on different categories. Three [21, 22, 26] presented the association the SUA level and CHD mortality based on an increase of 1 mg/dl in each level, and only one [32] represented it both ways. Nine studies [21, 23–28, 32, 33] reported the association between hyperuricemia and all-cause mortality. The majority of studies defined CHD mortality using the International Classification of Disease (ICD) codes from the hospital records or death certificates. All of the selected studies were assessed as high quality according to the NOS scale. The median NOS score of the studies selected was 9 (range from 7 to 9).

**Table 1:** Characteristics of studies included in the meta-analysis

Study	Year	Age (years)	Participants (%men)	Duration (years)	Hyperuricemia definition (mg/dl)	Confounding factors	Outcome definition	NOS score
CHA-W [21]	1989	35–64	6797 white women in USA	11.5	Per 1 mg/dl increase	Age, weight, smoking, DBP, cholesterol, antihypertensive drugs, ECG abnormalities	ICD-8 codes on death certificates	4/2/3
NHANES-I [22]	2000	25–74 (48.1)	5962 (45.6) non-institutionalized population in USA	16.4	M: 7.0; W: 5.6; Per 1 mg/dl increase	Age, race, BMI, cholesterol, smoking, alcohol, hypertension, DM, diuretic use	ICD-9 codes on death certificates, and/or a proxy interview	4/2/3
Tomita [23]	2000	25–60	49,413 male railroad workers in Japan	5.4	M: 6.5	Age	ICD-9 codes on health and pension records	3/1/3
Belgian study [24]	2001	25–74	9710 (53.9) subjects in Belgia	10	M: 7.0; W: 5.4	M:age, DBP, educational level, smoking, alcohol; W:age, cholesterol, SBP, BMI, smoking, alcohol, DM	ICD-9 codes on hospital records	4/2/3
KMIC [25]	2004	44.6 ± 8.7	22,698 men in Korea	9	M: 7.0	Age, DM, hypertension, cholesterol, smoking	Death certificates	4/2/3
Baibas [26]	2005	≥25	1198 (42) adults in rural Greece	14	Per 1 mg/dl increase	Age, village, educational level, weight, smoking, alcohol, SBP, blood glucose, cholesterol, triglycerides	ICD-9 codes on death certificates	4/2/3
Atomic bomb Study [27]	2005	20–89 (48.6)	10,615 (36.4) Japanese atomic bomb survivors	24.9	M: 7.0; W: 6.0	Age, BMI, smoking, alcohol, SBP, DM, cholesterol, histories of hypertension, kidney disease; malignant tumor; estimated radiation dose	ICD-7 to 10 codes on death certificates	3/2/3
Israeli male Study [28]	2006	49 ± 7	9125 men in Israel	23	M: 5.6	age, BMI, SBP, DM, smoking, LVH on ECG, cholesterol	ICD-9 codes on death certificates and hospital records	4/2/3
MRFIT [29]	2008	41–63	9105 men in USA	17	M: 7.0	Clinical center, age, BP, cholesterol, triglyceride, smoking, family history of AMI, aspirin and diuretic use, BMI	ICD-9 and 10 codes on death certificates	4/2/3
VHMPP-M [30]	2008	41.6	83,863 Austrian men	13.6	M: 6.7	Age, BMI, BP, cholesterol, triglycerides, glucose, smoking, year of examination	ICD-9 and 10 codes on death certificates; autopsy records	4/2/3
VHMPP-W [31]	2008	62.3 ± 8.8	28,613 elderly Austrian women	15.2	W: 5.4	Age, BMI, BP, cholesterol, triglycerides, glucose, smoking, occupational status, year of examination	ICD-9 and 10 codes on death certificates; autopsy records	4/2/3
Chinese cohort study [32]	2009	51.5 ± 11.5	90,393 (46.3) adults in Taiwan	8.2	M and W: 7.0; Per 1 mg/dl increase	Age, BMI, cholesterol, triglycerides, DM, hypertension, smoking, alcohol, sex	ICD-9 codes on death certificates	4/2/3
Puddu [33]	2014	35–74	2888 (44.1) adults from Gubbio in Italy	13.5	M and W: 7.0	Age, gender, SBP, cholesterol, smoking, BMI, blood glucose, e-GFR	ICD-9 codes on death certificates	4/2/3
NHANES-III [34]	2015	45.3	11,009 (45.9) adults in USA	14.5	M and W: 6.3	Age, sex, race, BMI, SBP, smoking, HDL, cholesterol, antihypertensive drug	ICD-10 codes on death certificates	4/2/3

*AMI* acute myocardial infraction, *BMI* body mass index, *BP* blood pressure, *CHA* Chicago Heart Association, *DM* diabetes mellitus, *DBP* diastolic blood pressure, *ECG* electrocardiograph, *e-GFR* estimated-glomerular filtration rate, *HDL* high-density lipoprotein, *ICD* international classification of disease, *KMIC* Korea Medical Insurance Corporation, *LVH* left ventricular hypertrophy, *M* men, *MRFIT* Multiple Risk factor Intervention Trial, *NHANES* National Health and Nutrition Examination Survey, *SBP* systolic blood pressure, *VHMPP* Vorarlberg Health Monitoring and Promotion Program, *W* women

### CHD mortality

**Fig. 2:**
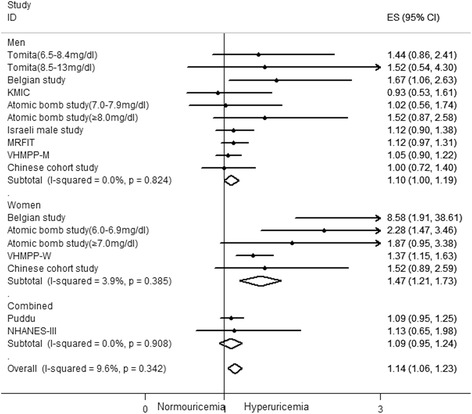
Forest plot of association between hyperuricemia and coronary heart disease mortality

The pooled multivariate adjusted RR for CHD mortality based on 11 studies [23–25, 27–34] was 1.14 (95 % CI: 1.06–1.23; Fig. 2). A slight heterogeneity between studies was noted (I^2^ = 9.6 %, *p* >0.05). No significant publication bias was present according to Begg’s and Egger’s test (both *P* values >0.05). The pooled adjusted RR for CHD mortality was 1.10 (95 % CI: 1.00–1.19) among men [23–25, 27–30, 32] and 1.47 (95 % CI: 1.21–1.73) among women [24, 27, 31, 32]. There was no evident heterogeneity between studies with respect to outcomes (I^2^ = 0.0 %, 3.9 %).

**Fig. 3:**
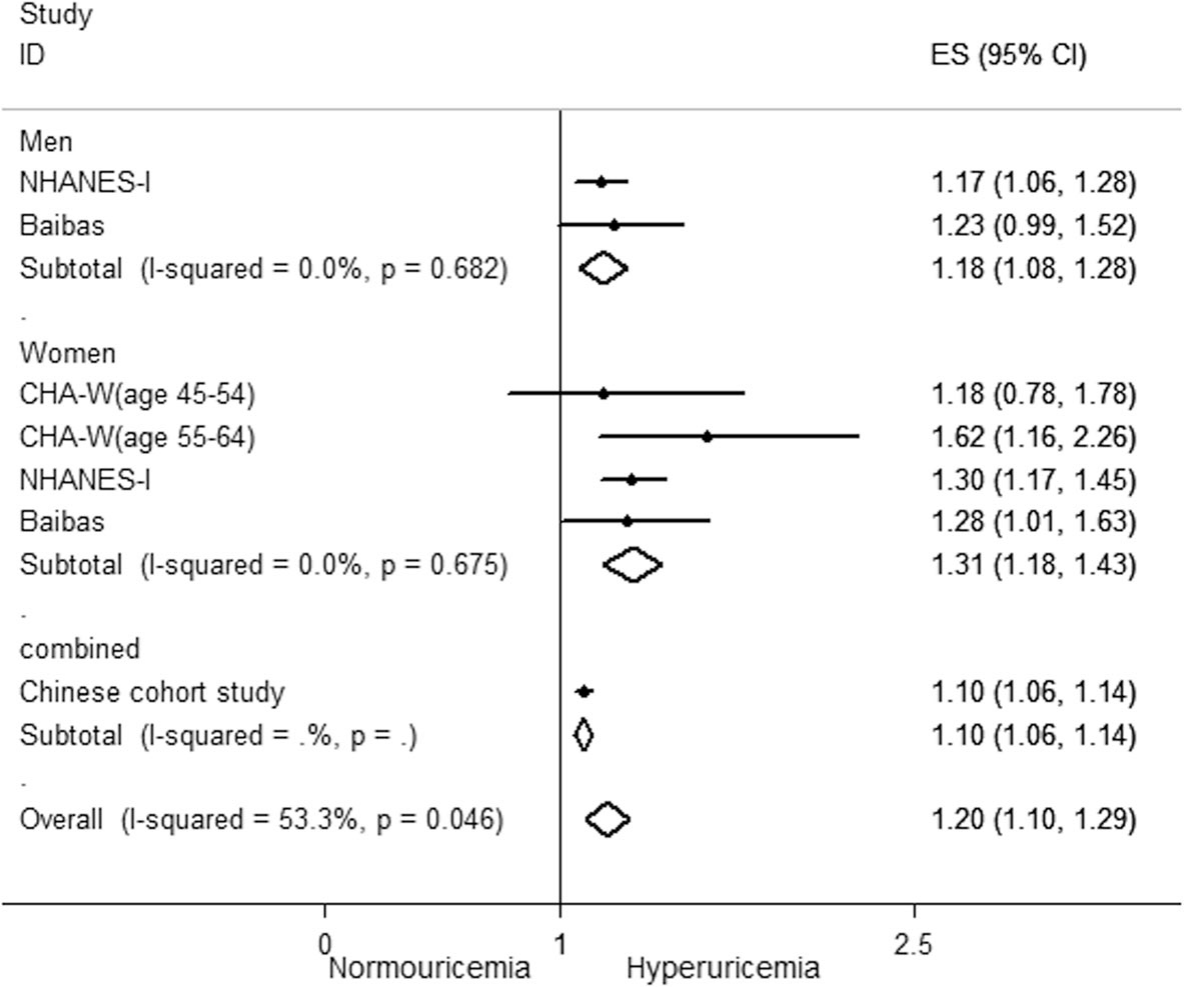
Forest plot of association between an increase of 1 mg/dl in serum uric acid level and coronary heart disease mortality

For each increase of 1 mg/dl in the SUA level, the pooled adjusted RR for CHD mortality based on four studies [21, 22, 26, 32] was 1.20 (95 % CI: 1.10–1.29; Fig. 3). Significant heterogeneity between studies was observed (I^2^ = 53.3 %, *p* <0.05). Subsequently, a sensitivity analysis was performed to identify the potential source of heterogeneity. Exclusion of one study [32] conducted by Chen et al. did not change the pooled results (RR: 1.31; 95 % CI: 1.15–1.31), but no evidence of heterogeneity was observed among the remaining studies (I^2^ = 0.0 %, *p* = 0.53). Further exclusion of any single study did not significantly alter the overall combined RR (data not shown). The pooled adjusted RR for each increase of 1 mg/dl in SUA was 1.18 (95 % CI: 1.08–1.28) among men [22, 26] and 1.31 (95 % CI: 1.18–1.43) among women [21, 22, 26]. No significant heterogeneity between studies was noted with respect to outcomes (I^2^ = 0.0 % for both genders).

### All-cause mortality

**Fig. 4:**
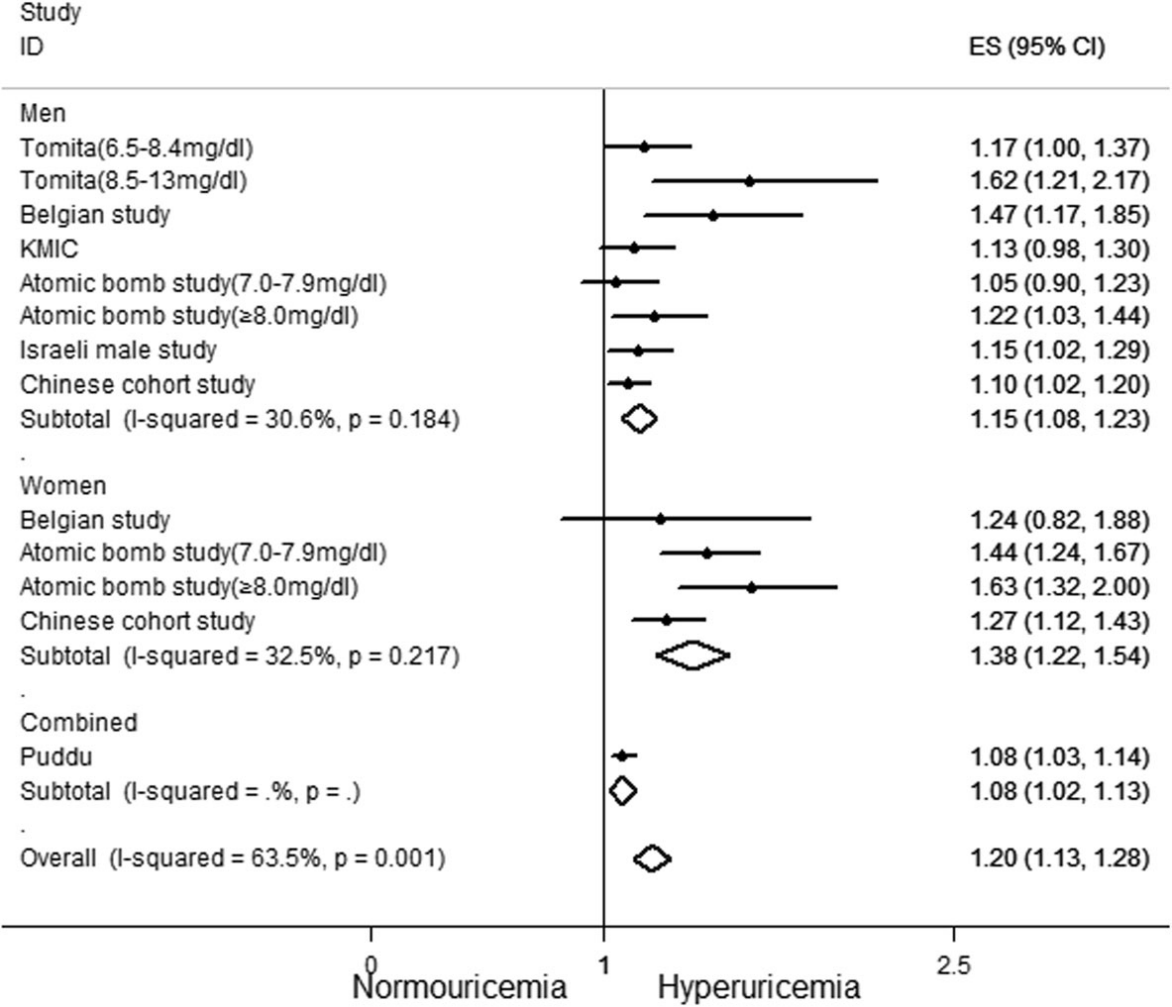
Forest plot of association between hyperuricemia and all-cause mortality

Nine studies [21, 23–28, 32, 33] reported outcomes on all-cause mortality. The pooled adjusted RR for all-cause mortality based on seven studies [23–25, 27, 28, 32, 33] was 1.20 (95 % CI: 1.13–1.28; Fig. 4). Significant heterogeneity between studies was observed (I^2^ = 63.5 %, *p* <0.01). There was a significant publication bias according to Begg’s test (*p* <0.05) and Egger’s test (*p* <0.01). Because the number of studies was less than ten, a multivariate meta-regression was not performed. We only performed a sensitivity analysis by excluding each study individually to identify the potential source of heterogeneity. Exclusion of any single study did not significantly alter the heterogeneity among the remaining studies. The pooled adjusted RR for all-cause mortality was 1.15 (95 % CI: 1.08–1.23) among men and 1.38 (95 % CI: 1.22–1.54) among women. There was slight heterogeneity between studies with respect to outcomes (I^2^ = 30.6 %, 32.5 %). Based on this, the heterogeneity among the seven studies may be related to gender.

**Fig. 5:**
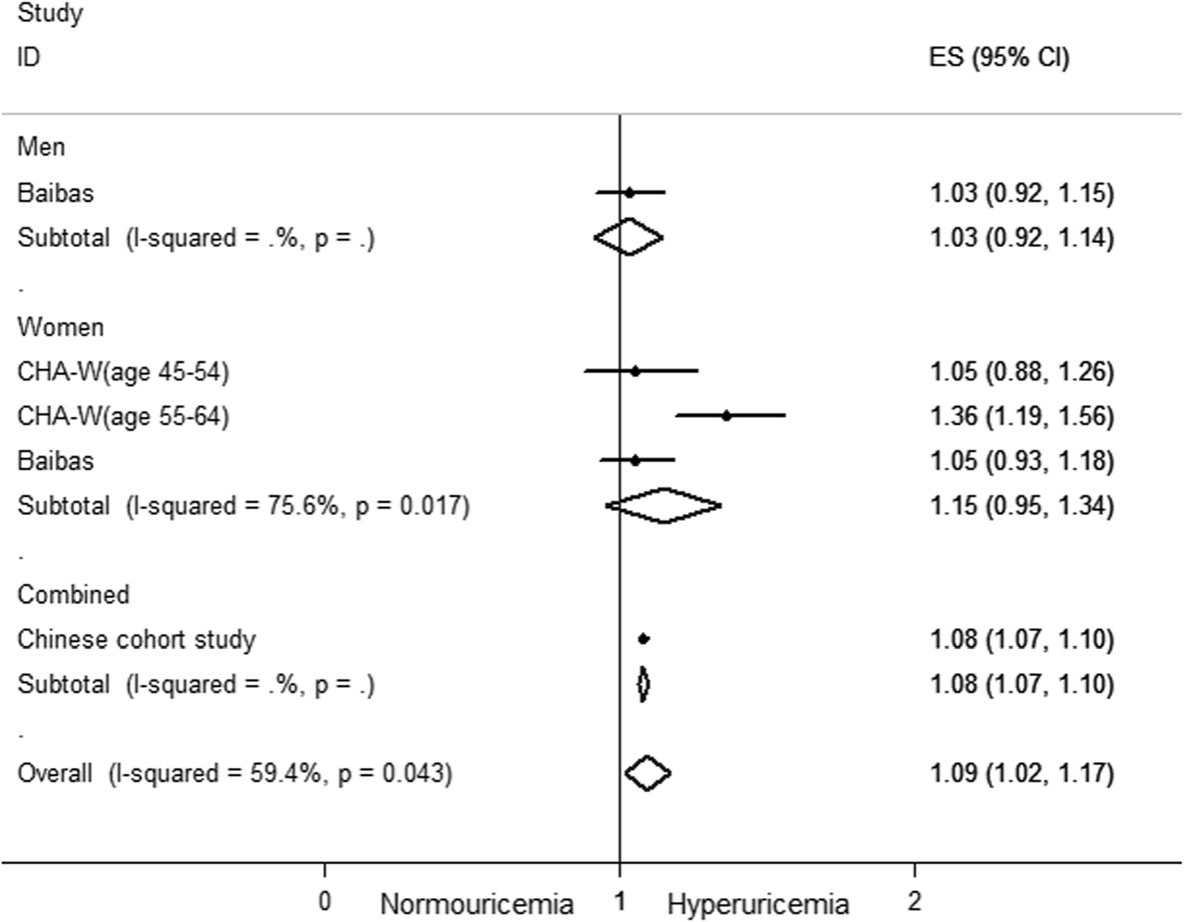
Forest plot of association between an increase of 1 mg/dl in serum uric acid level and all-cause mortality

For each increase of 1 mg/dl in the SUA level, the pooled adjusted RR for all-cause mortality based on three studies [21, 26, 32] was 1.09 (95 % CI: 1.02–1.17, Fig. 5). Significant heterogeneity between studies was observed (I^2^ = 59.4 %, *p* <0.05).

### Meta-regression

A multivariate meta-regression was performed to identify the predefined potential source of heterogeneity regarding CHD mortality. It demonstrated that the heterogeneity across studies was related to gender (*p* <0.05), rather than region, follow-up duration and sample size.

## Discussion

Our updated meta-analysis demonstrates that hyperuricemia is associated with a modest but statistically significant increased risk of CHD and all-cause mortality. For each increase of 1 mg/dl of SUA, the overall risks of CHD and all-cause mortality increased by 20 and 9 %, respectively. According to gender subgroup analyses, hyperuricemia increased the risk of CHD mortality in women (RR: 1.47; 95 % CI: 1.21–1.73) compared to men (RR: 1.10; 95 % CI: 1.00–1.19). The risk of all-cause mortality was greater for women.

Hyperuricemia has been correlated with hypertension, hyperlipidemia, diabetes, metabolic syndrome and renal disease, all of which could contribute to increased CHD and all-cause mortality. Over the past few decades, relevant studies [4, 5, 8, 13, 14, 21–37] have provided conflicting evidence regarding the association between hyperuricemia and CHD or all-cause mortality; therefore, whether hyperuricemia is an independent risk and causal factor for CHD mortality remains unclear. This phenomenon may be related to the differences in the enrolled populations, definition of hyperuricemia, outcomes studied, follow-up duration, sample size and statistical adjustment.

To further investigate the association between hyperuricemia and CHD or all-cause mortality, Zhao et al. [38] and Kim et al. [18] assessed it using a meta-analysis. The study conducted by Zhao et al. showed that elevated SUA increased the risk of cardiovascular mortality (RR: 1.37; 95 % CI 1.19–1.57) and all-cause mortality (RR: 1.24; 95 % CI: 1.09–1.42). The risk of cardiovascular mortality was more pronounced in women (RR: 1.35; 95 % CI: 1.06–1.72). However, the association between hyperuricemia and CHD mortality was not assessed independently. Our meta-analysis suggests that hyperuricemia is associated with all-cause mortality in both genders (Fig. 4), whereas Zhao et al. only observed this association for men (for women: RR 1.05; 95 % CI 0.79–1.39). The explanation for such findings may be related to the different inclusion criteria. Different from Zhao et al., we chose an inception cohort involving adults without CHD. In the other study, Kim et al. demonstrated that hyperuricemia was associated with an increased risk of CHD mortality (RR 1.16; 95 % CI 1.01–1.30), similar to our findings. However, there were several mistakes when the data (RR or 95%CI) were extracted from the original studies (Additional file 1: Tables S1 and S2) [24, 26, 30, 31], which may have altered the pooled outcomes. After correcting the mistake in their study, the overall pooled outcomes were not significantly changed. For the subgroup analysis, however, an increase of 1 mg/dl in the SUA level was associated with CHD mortality in both genders (Fig. 3), which is different from the previous meta-analysis [18] (RR 1.10, 95 % CI 0.96–1.24 among men; RR 1.17, 95 % CI 0.97–1.38 among women) but similar to ours. Therefore, this difference is because of the data extraction mistakes. Researchers found that patients with angiographically confirmed CHD with SUA levels in the upper quartile were five times more likely to die than those in the lowest quartile [39]. The risk of mortality increased by 26 % for each increase of 1 mg/dl in the SUA level and reflected the dose-response relationship between the SUA levels and all-cause mortality in patients with CHD. In a subsequent study in 2015, von Lueder et al. [40] investigated the relationship between SUA and clinical outcomes in subjects with acute myocardial infarction complicated by reduced left ventricular function or/and heart failure. Their study showed that SUA strongly and independently predict adverse outcomes, and the finding of dose dependent HR for all-cause and cardiovascular mortality through survival curves according to quartiles of baseline SUA. They concluded that the quantifcation of SUA could improve clinical risk stratifcation of patients with LV systolic dysfunction and/or HF following acute MI. Similarly, our meta-analysis is also in conformity to the results of the above two studies, but we suggests such a dose-response relationship in the general population, which may help to confirm the causal assiciation between HUA and CHD mortality from a different perspective.

Although it remains unclear as to the role that elevated SUA plays in CHD development and mortality, the evidence suggests the following possible mechanisms. First, several studies [41–45] suggested that hyperuricemia has a pathogenic role and predictive value in the development of hypertension. Therefore, a causal link to the development of hypertension is a plausible explanation for the possible increased cardiovascular risk in patients with hyperuricemia [46]. Second, increased SUA levels may encourage lipid peroxidation and promote the oxidation of low-density lipoprotein (LDL) cholesterol [47], which may play a role in the development of atherosclerosis [48] and would also explain its association with CHD [49]. Interestingly, because human atherosclerosis plaques contain more UA than normal artery walls, researchers propose that SUA may have a direct role in the atherosclerosis process [50]. Third, hyperuricemia may induce endothelial dysfunction, which is predicted to promote the early development of atherosclerosis and precede plaque formation [51]. The deposition of urate crystals on the vessel wall could cause an inflammatory reaction to then directly injure the vascular intima and ultimately activating the platelet and blood coagulation system. Finally, hyperuricemia also promotes thrombosis [52, 53] and activates monocyte chemotactic protein-1 [46], an important chemokine in atherosclerosis.

Greater attention has been paid to whether urate-lowing therapy improves cardiovascular outcomes. Hyperuricemia is frequently encountered in hypertensive patients. Patients with hyperuricemia and hypertension are associated with a 3- to 5-fold increase in CHD compared to patients with normal SUA levels [54]. LIFE is the first study to demonstrate that reducing the SUA levels is associated with a reduction of cardiovascular events in hypertensive patients [55]. Allopurinol, a xanthine inhibitor, is frequently used in hyperuricemic patients to reduce the SUA level. A meta-analysis of 10 studies showed that allopurinol is associated with a small but significant reduction in blood pressure [56]. High-dose allopurinol therapy may prolong the time to chest pain during exercise and improve endothelial dysfunction in patients with stable angina pectoris [57]. These effects of allopurinol may be valuable for reducing future cardiovascular mortality. Encouragingly, a prospective cohort study (*n* = 7135) demonstrated that high-dose allopurinol treatment is associated with a lower risk of cardiovascular events and mortality [58]. Although previous studies have not yet provided direct evidence that urate-lowering therapy reduces the risk of CHD mortality in hyperuricemic patients, the studies discussed above provided some positive data. Hence, further research is necessary.

Several limitations of this meta-analysis should be acknowledged. First, although a multivariable adjustment was conducted in most of the included studies, confounding effects from other unadjusted risk factors may exist. Notably, the majority of the considered studies were not adjusted for renal functionality or diuretics or purine and fructose intake, which significantly influence the SUA level. In particular, renal functionality is a main determinant of CHD and its mortality. So more studies with subject-restriction to only those participants with normal eGFR are needed to conduct in the future, only by this, the causal relationship between hyperuricemia and risk of CHD mortality could be accurately detected. Second, our results may be less convincing because the SUA level may also be associated with other organ damage, such as heart failure or IMT, or to the development of other relevant diseases, such as type 2 diabetes [59]. Third, it’s important to note that there was a significant publication bias with regards to all-cause mortality. We attempted to minimize publication bias by searching electronic databases with no language restriction; however, because many researchers didn’t focus on all-cause mortality and report it as a primary or secondary outcome in related studies, and studies with negative results and that are written in non-English languages are less likely to be published, publication bias still exists. Despite these limitations, our study has several strengths. This meta-analysis is based on large prospective cohort studies with a long follow-up period in many different areas. Most of the studies included in our meta-analysis reported the adjusted RR. We assessed the quality of individual studies using the Newcastle-Ottawa Scale, which shows that all of the studies were of high quality, making our results more reliable. In addition, compared to the previous meta-analysis [18], we included the latest studies and corrected the data errors extracted from the four papers (Additional file 1: Tables S1 and S2) [24, 26, 30, 31] that we included. Because of this, our statistical subgroup analyses suggested that regardless of hyperuricemia or an increase of 1 mg/dl in the SUA level, both are associated with CHD mortality in both genders. Furthermore, we conducted a multivariate meta-regression analysis on the log-transformed scale of RR to explore the impact of the study characteristics.

## Conclusions

In conclusion, our meta-analysis suggests that hyperuricemia may modestly increase the risk of CHD and all-cause mortality. Because there are safe and effective methods to reduce SUA levels, future studies should focus on the role of urate-lowering therapy for reducing CHD mortality.

## Additional file

Additional file 1: Table S1. The erroneous and correct data for the association between hyperuricemia and CHD mortality. **Table S2.** The erroneous and correct data for the association between an increase of 1 mg/dl in serum uric acid level and CHD mortality. (DOC 24 kb)

## Abbreviations

CHD: Coronary heart disease
HR: Hazard ratio
ICD: International Classification of Disease
LDL: Low-density lipoprotein
MOOSE: Meta-analysis of Observational Studies in Epidemiology
RR: Relative risk
SUA: Serum uric acid
