# Supplementary material for: Hyperuricemia and coronary heart disease mortality: a meta-analysis of prospective cohort studies
Source: BMC Cardiovasc Disord. 2016 Oct 28;16:207. doi: 10.1186/s12872-016-0379-z (PMC5084405; doi:10.1186/s12872-016-0379-z)
Supplement: Additional file 1: Table S1. — The erroneous and correct data for the association between hyperuricemia and CHD mortality. Table S2. The erroneous and correct data for the association between an increase of 1 mg/dl in serum uric acid level and CHD mortality. (DOC 24 kb) [file 12872_2016_379_MOESM1_ESM.doc]

Table S1 The erroneous and correct data for the association between hyperuricemia and CHD mortality

Gender Study Erroneous Data Correct Data

Male Belgian study[25] 1.62(1.06, 2.50) 1.67(1.06, 2.63)

VHMPP-M[31] 1.02(0.81, 1.27) 1.05(0.90, 1.22)

Female VHMPP-W[32] 1.58(1.19, 2.10) 1.37(1.15, 1.63)

Table S2 The erroneous and correct data for the association between an increase of 1 mg/dl in serum uric acid level and CHD mortality

Gender Study Erroneous Data Correct Data

Male Baibas[27] 1.03(0.92, 1.15) 1.23(0.99, 1.52)

Female Baibas[27] 1.05(0.93, 1.18) 1.28(1.01, 1.63)

CHD=coronary heart disease
